# A Microflow Cytometry-Based Agglutination Immunoassay for Point-of-Care Quantitative Detection of SARS-CoV-2 IgM and IgG

**DOI:** 10.3390/mi12040433

**Published:** 2021-04-14

**Authors:** Jianxi Qu, Mathieu Chenier, Yushan Zhang, Chang-qing Xu

**Affiliations:** 1 School of Biomedical Engineering, McMaster University, 1280 Main Street West, Hamilton, ON L8S 4L8, Canada; quj@mcmaster.ca (J.Q.); zhang749@mcmaster.ca (Y.Z.); 2 Department of Engineering Physics, McMaster University, 1280 Main Street West, Hamilton, ON L8S 4L8, Canada; chenim1@mcmaster.ca

**Keywords:** SARS-CoV-2 IgM, SARS-CoV-2 IgG, microflow cytometry, agglutination immunoassay, point-of-care

## Abstract

A rapid, sensitive and simple microflow cytometry-based agglutination immunoassay (MCIA) was developed for point-of-care (POC) quantitative detection of SARS-CoV-2 IgM and IgG antibodies. The antibody concentration was determined by using the transit time of beads aggregates. A linear relationship was established between the average transit time and the concentration of SARS-CoV-2 IgM and IgG, respectively. The limit of detection (LOD) of SARS-CoV-2 IgM and IgG by the MCIA measurement are 0.06 mg/L and 0.10 mg/L, respectively. The 10 µL sample consumption, 30 min assay time and the compact setup make this technique suitable for POC quantitative detection of SARS-CoV-2 antibodies.

## 1. Introduction

Despite the COVID-19 pandemic persisting for more than a year, there is still a need for a fast and sensitive antibody test to detect SARS-CoV-2 infections. Unlike diagnostic tests, antibody or serological tests are not intended to detect infections at their onset. Rather, they detect if an individual has produced antibodies in response to a specific foreign body, in this case, the SARS-CoV-2 virus. Since seroconversion occurs some time after initial infection, antibody tests are not intended to replace diagnostic testing methods, namely PCR tests [1]. Rather, antibody tests are intended to complement diagnostic testing and identify individuals previously infected with SARS-CoV-2. Further, due to their ability to detect past infections, antibody tests can be deployed for epidemiological purposes to monitor the spread of COVID-19 [2].

POC antibody tests are serological tests that do not require the use of laboratory technicians or facilities to provide results [3]. POC testing, unlike intensive laboratory methods, is timely and convenient. A fast and simple POC antibody test could allow for mass screening, assist in contact tracing and help monitor the spread of COVID-19 [2,4]. Further, a POC antibody test has the advantage of being accessible, allowing it to be used at health care facilities, at home, or in the field without the equipment required by some laboratory techniques [3]. For a POC antibody test to be an effective alternative to current laboratory methods, it should be fast, quantitative, portable, inexpensive, easy to operate and require small sample volumes while remaining sensitive and specific.

Currently, enzyme-linked immunosorbent assays (ELISAs) and lateral flow immunoassays (LFIAs) are two common methods used to detect SARS-CoV-2 antibodies. ELISAs must be performed by skilled operators, have long incubation times and require laboratory equipment to quantify results, making them impractical for POC environments [5]. LFIAs, although suitable for POC environments, are not traditionally quantifiable and can provide ambiguous results [6]. Tests of both formats have reported wide ranges of sensitivity and specificity and have been evaluated under high risk of patient selection bias, making it unclear if they are suitable for mass testing [2,7,8,9].

Other testing formats have been proposed for the detection of SARS-CoV-2 antibodies including flow cytometry. Grzelak et al. developed a technique to detect anti-spike (S) protein antibodies by expressing the S protein on the surface of human embryonic kidney 293T cells. The cells were then incubated with human serum samples and fluorescent anti-IgG or anti-IgM before being evaluated by flow cytometry [8]. Horndler et al. developed a similar method by expressing the SARS-CoV-2 S protein on the human leukemic T cell line [10]. These methods may not be suitable for POC detection, however, as they require flow cytometry equipment and cell cultures that must remain stable to provide sensitive testing. Fluorescent microsphere immunoassays (FMIAs) have also been developed for the detection of SARS-CoV-2 antibodies, with one method being granted an emergency use authorization (EUA) by the Food and Drug Administration (FDA). Typically, in the FMIA methods, SARS-CoV-2 antigens are bound to microbeads. These complexes are then mixed with serum samples and fluorescent secondary antibodies to produce a quantifiable signal. Cameron et al. describe an FMIA for the detection of anti-N, S and receptor-binding domain (RBD) antibodies [11]. These formats are currently destined for laboratory use only as they require large flow cytometry equipment.

In this work, a fast and sensitive MCIA suitable for the POC quantitative detection of SARS-CoV-2 IgM and IgG was proposed. Microbeads were conjugated to anti-IgM and anti-IgG antibodies and incubated with SARS-CoV-2 antibodies. SARS-CoV-2 S protein was then added to cause microbeads to agglutinate. Using microflow cytometry, transit time was measured and used to distinguish monomers from larger aggregates. The relationship between the average transit-time of the aggregates and certain concentrations of SARS-CoV-2 IgM and IgG was studied. A linear correlation was found. This work also discussed the potential application of the MCIA for the POC detection and quantification of SARS-CoV-2 IgM and IgG.

## 2. Materials and Methods

**Figure 1:**
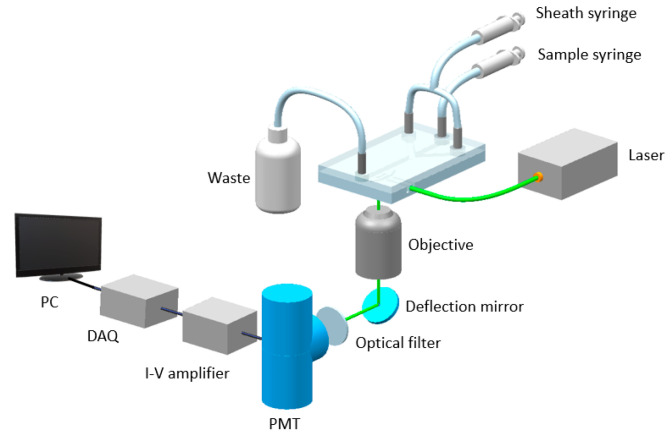
A diagram of the microfluidic platform for SARS-CoV-2 antibody detection.

Figure 1 shows the microfluidic platform used to measure SARS-CoV-2 antibodies. Light emitted from a 532 nm green laser was coupled into the input waveguide of a microflow cytometry device and then focused into the center of a microfluidic channel through an embedded lens system. The microfluidic chip was made by the photolithography. It consists of four layers: Pyrex glass layer, SU-8 photoresist layer, polydimethylsiloxane (PDMS) layer and a glass layer. The bottom Pyrex glass layer was used as the substrate. The SU-8 layer, with a thickness of 50 µm, was patterned with microfluidic channels by photolithography. PDMS was used to seal the SU-8 layer to form microfluidic channels. The top glass layer was used to fix the metal pins which are used as the inlets and outlets of the chip. The cross-sectional area of the microchannel is 50 µm × 100 µm (height × width). The dimensions of the chip are approximately 3 cm × 2 cm × 0.5 cm (length × width × height). Assay mixtures from the immune reaction were introduced into the microfluidic channel by an external syringe pump and hydrodynamically focused into a narrow stream by two sheath flows. The aggregates passing through the interrogation region (the intersection of the laser beam and the microchannel) interacted with the coupled laser beam, resulting in scattered light. The scattered light was collected by an objective lens which was mounted perpendicular to both the light beam and the microchannels, then deflected by a mirror. The deflected light passed through a band pass filter and then was amplified by a photomultiplier tube (PMT). A current-to-voltage amplifier was used to convert current signals from the PMT into amplified voltage signals. The amplified voltage signals were digitized by a data acquisition board (DAQ) and then processed by a custom LabVIEW program with a personal computer. The details of the microflow cytometry device were presented in a previous publication [12]. It is worth noting that, since both the microchannel and optical lens are integrated in the microflow cytometry device, the microfluidic platform shown in Figure 1 can be easily packaged into a small box by employing an advanced, off-the-shelf micro-PMT.

**Figure 2:**
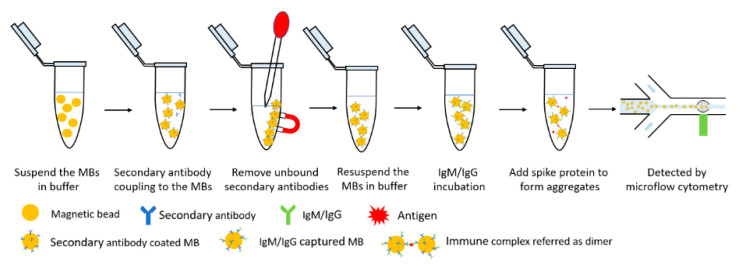
Schematic illustration of the MCIA for the quantitative detection of SARS-CoV-2 IgM/IgG.

Figure 2 presents the immune reaction steps of the MCIA. The protocols for the detection of SARS-CoV-2 IgM and IgG are the same, except for the secondary antibody used. The following protocols will describe the SARS-CoV-2 IgM measurement and is a model to illustrate the immunoassay. The first step is secondary antibody coupling. Goat anti-human IgM secondary antibodies (31136, Invitrogen, Thermo Fisher Scientific, Rockford, MD, USA) were coupled to Dynabeads M-270 Epoxy (14301, Invitrogen, Thermo Fisher Scientific, Carlsbad, CA, USA) magnetic beads (MBs) according to the user manual. The lyophilized powder of MBs has a concentration of 6.7 × 10^7^ beads/mg. The MBs need to be washed before the antibody coupling procedure. A total of 5 mg of lyophilized beads were weighed out and resuspended in 1 mL of 0.1 M sodium phosphate buffer (PB) (P5244, Millipore Sigma, Oakville, ON, Canada). The MBs were incubated for 10 min with tilting and rotation and then applied to a magnet. The supernatant was removed by a pipette. The MBs were suspended in 1 mL of PB again and vortexed for 30 s. The MBs were ready to couple to secondary antibodies after removing the supernatant. A total of 5 mg of MBs were used to couple approximately 100 µg of secondary antibody as recommended by the user manual. The concentration of goat anti-human IgM secondary antibodies was 2.32 mg/mL. Therefore, the volume of secondary antibody needed for the coupling procedure was approximately 43 µL. The washed MBs were suspended in 43 µL of PB with mixing. Then, 43 µL of goat anti-human IgM secondary antibody was added into the solution with thoroughly mixing. An equal volume, 43 µL of 1 M ammonium sulfate buffer was added into the solution with mixing and then the MB solution was incubated at 37 °C for 24 h. After the incubation, the MBs were separated from the solution using the magnet separation procedure. Then, the MBs were washed with 1 mL phosphate buffered saline (PBS, pH = 7.4) four times. The MBs were, finally, suspended in 330 µL of PBS with a concentration of 1 × 10^9^ beads/mL. The secondary antibody coated MBs were stored at 4 to 8 °C with 0.02% (*w*/*v*) sodium azide (08591, Sigma-Aldrich, Millipore Sigma, Oakville, ON, USA) to provide a long shelf-life for the MCIA antibody test. The recommended ratio between the secondary antibodies and the MBs from the user manual was used in this study and the antibody coupling efficiency has been verified by coupling MBs to anti-human IgM secondary antibody-HRP instead of the goat anti-human IgM. This provided evidence that the secondary antibodies were successfully captured by the MBs using the recommended ratio. For the antibody test, the concentration of the secondary antibody conjugated MBs was optimized and should be higher than 5 × 10^6^ beads/mL. The storage conjugated MBs were washed with PBS containing 0.1% bovine serum albumin (BSA) (15561020, Invitrogen, Thermo Fisher Scientific, Carlsbad, CA, USA) once and then diluted by PBS to the desired concentration. A total of 10 µL of diluted conjugated MBs were resuspended in a 600 µL centrifuge vial. 10 µL of SARS-CoV-2 RBD IgM humanized coronavirus monoclonal antibodies (MBS355900, MyBioSource, Inc., San Diego, CA, USA) were added into the vial and mixed with a pipette tip for 30 s. After a 25 min incubation, during which SARS-CoV-2 IgM antibodies were captured by the IgM secondary antibodies, the conjugated MBs were sonicated for 10 s and then 10 μL of SARS-CoV-2 spike S1-His recombinant protein (40591-V08B1, Sino Biological Inc., Beijing, China) with a concentration of 25 mg/L was added into the vial and mixed for 30 s. The aggregates were formed in this incubation procedure. After a 3 min incubation, the assay mixtures were introduced into the microflow cytometer by the external syringe pump. The flow rate for sample and sheath flows were set as 200 µL/h and 800 µL/h, respectively. A total of 2 min of data were collected and analyzed by the LabView program. For the SARS-CoV-2 IgG test, goat anti-rabbit IgG (H + L) antibodies (31210, Invitrogen, Thermo Fisher Scientific, Carlsbad, CA, USA) were coupled to the MBs instead. SARS-CoV-2 spike S1 antibodies (40150-R007, Sino Biological, Inc., Beijing, China) were tested by the MCIA. It is worth mentioning that the described immune reaction step of the MCIA can be easily automated, which is important for its POC applications.

## 3. Results and Discussion

The MCIA is derived from the particle counting immunoassay with the principle that agglutination caused by an immune reaction decreases the number of free particles in assay mixtures [13]. Microflow cytometry is a competitive tool for particle counting due to its compacted size, low reagent consumption and relatively high sensitivity [14]. Generally, light intensity of the side scattered light from microflow cytometry is analyzed. Large sizes of beads or aggregates tend to result in higher scattered light intensities and thus can be used to discriminate different sizes of beads. However, the relatively high deviations of scattered light intensity affect the detection sensitivity [15]. Additionally, using light intensities to discriminate between the sizes of single beads and aggregates is unfeasible. In this proposed method, transit time was used to discriminate between the different sizes of beads and aggregates. The time it took for beads to pass through the interrogation region was defined as the transit time—the interval between the intercepts of the signal pulse and the threshold. The threshold was the standard deviation of the background noise, which was used to differentiate signals from noise. Details regarding how to use the average transit time to analyze data can be found from our previous publication [16]. 

**Figure 3:**
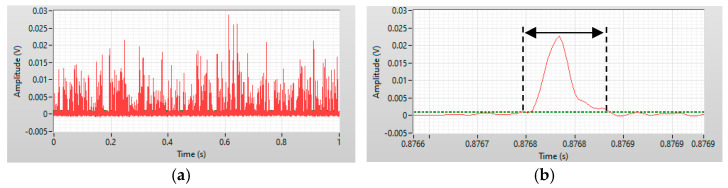
(**a**) One second of raw data showing the side scattered light signal obtained from a 0.6 mg/L SARS-CoV-2 IgM sample. (**b**) A detailed view of a side scattered light signal showing the definition of the transit time.

Figure 3 shows the side scattered light signals of a 0.6 mg/L SARS-CoV-2 IgM sample recorded by the custom program. The *x*-axis is the time in milliseconds and the *y*-axis represents the amplitude of the signal. Red solid lines are waveforms of the signals. Peaks in Figure 3a were produced when aggregates of MBs passed through the interrogation region. Figure 3b shows the definition of the transit time. The signal was distinguished from the noise by the threshold, which is the green dashed line. The threshold value was the standard deviation of the background noise. 

**Figure 4:**
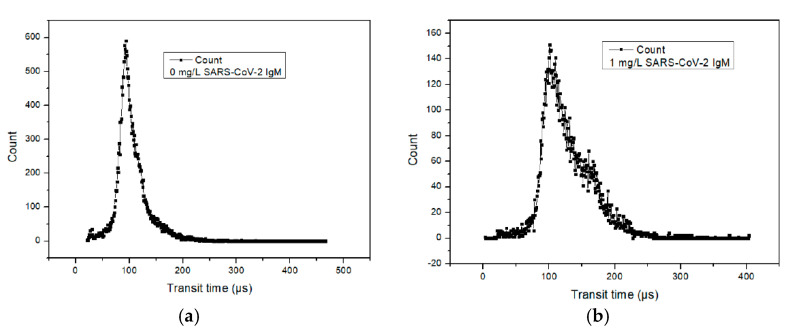
Transit time distribution achieved from the detection of (**a**) negative sample (PBS) and (**b**) 1 mg/L SARS-CoV-2 IgM.

Compared with monomers, aggregates have longer transit times. Figure 4a shows the transit time distribution obtained from a negative control in which PBS was used as the sample and Figure 4b shows a sample containing 1 mg/L SARS-CoV-2 IgM. The *x*-axis in Figure 4 is the transit time and the *y*-axis in Figure 4 is the number of monomers and aggregates with different transit times counted by the microflow cytometer. Each solid square represents the number of single MBs or aggregates. The decrease in the proportion of monomers and the increase of aggregates is obvious in terms of the transit time. The distribution of the transit time for the negative control in Figure 4a is tapered and symmetrical because most of the beads in the assay mixtures were monomers. The distribution of the transit time for the 1 mg/L SARS-CoV-2 IgM in Figure 4b has a peak that is short and wide. Compared with the distribution of the transit time of the negative control, an obvious peak shoulder was presented in the 1 mg/L SARS-CoV-2 IgM group, which was attributed to increased aggregate formation caused by the immune reaction. Other detected samples containing different concentrations of SARS-CoV-2 IgM and IgG also showed similar distributions of the transit time as shown in Figure 4b. By calculating the average transit time, a relationship can be established between the average transit time and the concentration of SARS-CoV-2 antibodies.

**Figure 5:**
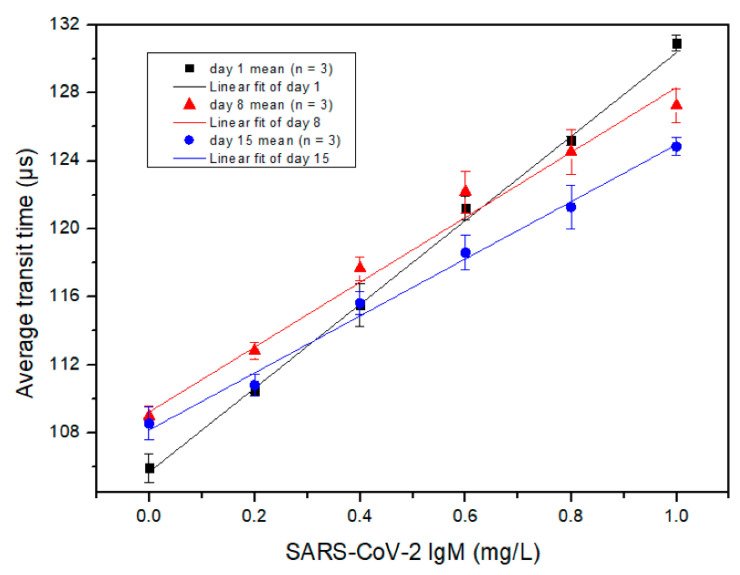
The measured relationship between the average transit time and the concentration of SARS-CoV-2 IgM detected at different days of secondary antibody conjugated MB storage. Each symbol is the mean value of three detections from duplicated samples. Error bars are the standard deviation of the three detections.

Figure 5 shows the measured correlation between the average transit time and the SARS-CoV-2 IgM concentration with linear fittings, determined by the MCIA. The *x*-axis is the concentration of SARS-CoV-2 IgM and the *y*-axis is the average transit time. The stored goat anti-human IgM secondary antibody coated MBs were used to detect SARS-CoV-2 IgM on different days. Different coloured symbols, including black squares, red triangles and blue circles, are used in Figure 5 to represent testing results obtained from days 1, 8 and 15, respectively. Each symbol and error bar are the mean value of three detections from duplicated samples and the standard deviation of the three detections, respectively. As shown in Figure 5, the average transit time has a strong linear relationship with the concentration of SARS-CoV-2 IgM when the test is performed on different days, although the slope of the linear curve decreases with the increase of anti-IgM conjugated MBs storage time. Therefore, the performance of the conjugated MBs decreased with the elapse of conjugated MB storage time. For days 1, 8 and 15, the R^2^ was 0.9984, 0.9982 and 0.9941, respectively. The LOD of SARS-CoV-2 IgM for the MCIA was determined as 3.3 times the residual standard deviation of the regression line divided by the slope. Day 1 data were used to calculate the LOD, which was determined to be 0.06 mg/L. Iyer et al. reported a LOD of 0.28 mg/L for anti-RBD SARS-CoV-2 IgM for their in-house ELISA method, which is comparable to the LOD achieved by the MCIA [17]. Additionally, the average transit time deviated from the linear relationship as the concentration of SARS-CoV-2 IgM surpassed 1.0 mg/L due to the prozone effect [18]. Therefore, it is necessary to keep the concentration of SARS-CoV-2 IgM below 1.0 mg/L to avoid false positive results. This may be done by diluting biological samples.

**Figure 6:**
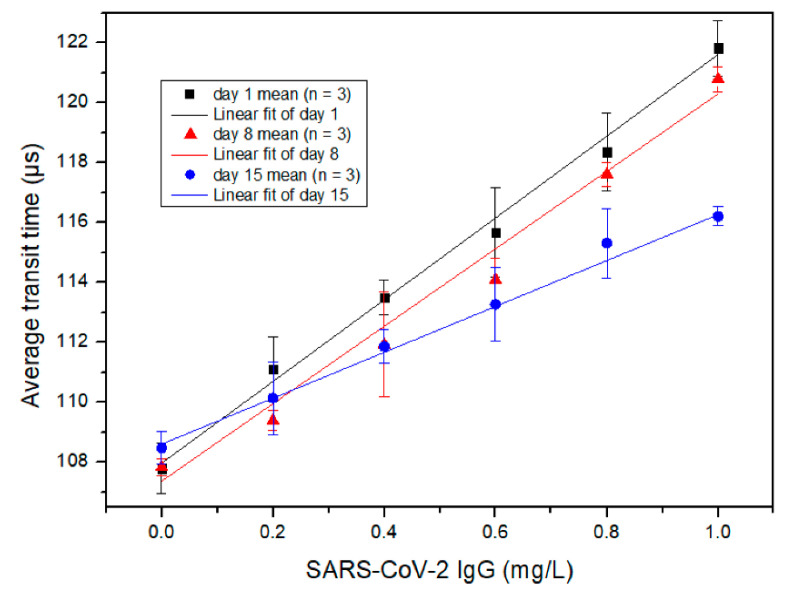
The measured relationship between the average transit time and the concentration of SARS-CoV-2 IgG detected at different days of secondary antibody conjugated MB storage. Each symbol is the mean value of three detections from duplicated samples. Error bars are the standard deviation of the three detections.

Figure 6 shows the measured correlation between the average transit time and the concentration of SARS-CoV-2 IgG, determined by the MCIA. The *x*-axis is the concentration of SARS-CoV-2 IgG antibody and the *y*-axis is the average transit time. Goat anti-rabbit IgG (H + L) secondary antibody coated MBs with different storage times were used for the detection of SARS-CoV-2 IgG. Different coloured symbols including black squares, red triangles and blue circles, are used in Figure 6 to represent testing results obtained from days 1, 8 and 15, respectively. Each symbol and error bar represent the mean value of three detections from duplicated samples and the standard deviation of the three detections, respectively. Similar to the results for SARS-CoV-2 IgM shown in Figure 5, the average transit time has a strong linear relationship with the concentration of SARS-CoV-2 IgG on different days of storage, although the slope of the linear curve decreases with the increase of conjugated MBs storage time. The performance of the conjugated MBs decayed with the elapse of time, but the R^2^ for each curve was consistent; for days 1, 8 and 15, the R^2^ was 0.9981, 0.9963 and 0.9987, respectively. The LOD of SARS-CoV-2 IgG for the MCIA was determined as 3.3 times the residual standard deviation of the regression line divided by the slope. Day 1 data were used to calculate the LOD, which was determined to be 0.10 mg/L. Iyer et al. reported a LOD of 0.04 mg/L for anti-RBD SARS-CoV-2 IgG for their in-house ELISA method, which is comparable to the LOD achieved by the MCIA [17]. In addition, the average transit time deviated from the linear relationship as the concentration of SARS-CoV-2 IgG exceeded 1 mg/L due to the prozone effect [18]. Therefore, it is necessary to keep the concentration of SARS-CoV-2 IgG below 1.0 mg/L for the MCIA. Again, this may be accomplished by dilution.

According to the results presented in Figure 5 and Figure 6, the slopes of the linear curves for the SARS-CoV-2 IgM measurements were greater than those for SARS-CoV-2 IgG. This is likely due to the pentameric structure of IgM, which increases the probability of aggregates to form. 

To test the performance of the MCIA for its practical application, a certain number of SARS-CoV-2 antibodies diluted by human serum (H4522, Human serum, Sigma-Aldrich, Oakville, ON, Canada) were spiked into the samples and then tested by the MCIA. The recovery index was calculated by the following equation, where *C_spiked sample_* is the concentration of the sample after the addition of a concentrated standard, which was diluted by the human serum. *C_unspiked sample_* is the concentration of the sample prepared with PBS. *C_added_* is the concentration of the added standard in the whole volume.

$$\%\;\mathrm{Recovery}\;\mathrm{index}=\frac{C_{spiked\;sample}-C_{unspiked\;sample}}{C_{added}}\times 100\%$$

For the detection of SARS-CoV-2 IgM, 16 µL samples prepared by PBS were split into two equals. One was added with 2 µL of deionized water and the other was added with 2 µL of the concentrated standard of SARS-CoV-2 IgM. Both samples were measured by the MCIA. The concentrated standard was diluted by the human serum sample and *C_added_* was calculated to be 0.4 mg/L. *C_unspiked sample_* was diluted by PBS to a concentration of 0.4 mg/L. In this case, *C_spiked sample_* should be 0.8 mg/L, provided that the recovery index was 100%. After measurement of the samples, the concentrations of the samples were obtained from the calibration curve of the SARS-CoV-2 IgM day 1 measurement (Y = 24.964 × X + 105.73), which was shown in Figure 5. The experiments were repeated once and the averages were used to calculate the recovery index, which was determined to be 96.85%. The recovery index of SARS-CoV-2 IgG was calculated in a similar manner to SARS-CoV-2 IgM. The calibration curve of SARS-CoV-2 IgG day 1 detection in Figure 6 was Y = 13.442 × X + 107.99. The calculated recovery index for SARS-CoV-2 IgG was 98.98%. From a practical point of view, both recovery indexes were acceptable.

The proposed MCIA can be used for the quantitative detection of SARS-CoV-2 antibodies and other kinds of antibodies as well. Compared with other POC antibody tests such as LFIAs, the MCIA can provide quantitative results instead of qualitative ones. The quantitative measurement of antibodies provides more information regarding the degree of the immune response, which may be helpful for monitoring the progression of COVID-19. The assay time of the MCIA is about half an hour, which meets the requirement of a POC test. The 10 µL sample volume consumption of the MCIA is comparable to most POC devices. Although the cost per analysis of the MCIA is dependent on the reagents needed, the MCIA is based on the microflow cytometry format, so the inherent advantages of microflow cytometry, such as cost-efficiency, also exist for the MCIA. The linear range of antibody concentrations detected by the MCIA is restricted by the prozone effect, but dilution can be performed when the concentration of the sample exceeds this range. Furthermore, it is important to note that there is about a 75% similarity between the genomes of SARS-CoV and SARS-CoV-2 [19]. The MCIA for SARS-CoV-2 detection did have cross-reactivity with IgG from SARS-CoV based on the results from our experiments. 

## 4. Conclusions

A MCIA has been developed for the POC quantitative detection of SARS-CoV-2 IgM and IgG antibodies. A linear relationship has been established between the average transit time and the concentration of SARS-CoV-2 antibodies up to 1.0 mg/L. 0.06 mg/L and 0.10 mg/L are the LODs for SARS-CoV-2 IgM and IgG, respectively. These values are comparable to those reported for in-house ELISAs, indicating that the device could adequately perform if tested against serum samples. The proposed protocol has demonstrated to be rapid, simple and sensitive for the POC quantitative measurement of SARS-CoV-2 antibodies. The 30 min assay time, 10 µL sample consumption, relatively simple procedures and compact core devices make the MCIA a promising tool for the POC detection of SARS-CoV-2 IgM and IgG. Furthermore, the developed protocol is readily adaptable to be applied for the detection of other antibodies. However, there are still some limitations of this study. The detection ranges of SARS-CoV-2 IgM and IgG were not large enough for the method to be conceivably performed without dilution of serum samples. The manual steps for the immune reaction need to be integrated into the MCIA to improve the detection efficiency. Further investigation of this study should focus on reducing the mentioned limitations and testing the sensitivity and specificity of the MCIA performance using serum specimens from individuals who have been infected with SARS-CoV-2. 

## Data Availability

Data available on request due to restrictions of privacy.
